# Identification of *Legionella feeleii* Cellulitis

**DOI:** 10.3201/eid1701.101346

**Published:** 2011-01

**Authors:** Severine Loridant, Jean-Christophe Lagier, Bernard La Scola

**Affiliations:** Author affiliations: Université de la Méditerranée, Marseille, France (S. Loridant, J.-C. Lagier, B. LaScola);; Hôpital Nord, Marseille (J.-C. Lagier)

**Keywords:** Bacteria, Legionella feeleii, cellulitis, skin infections, je ne sais pas, shell vial culture, cutaneous infections, France, letter

**To the Editor:** In general, reports of extrapulmonary *Legionella* spp. infections are scarce. For example, *L. micdadei* infection was found with the following manifestations: a mass on the left side of the neck and low-grade fever in a healthy 9-year-old girl (*1*); multiple liver and lung abscesses in a 7-year-old girl with acute lymphoblastic leukemia who had undergone allogeneic cord blood transplantation (*2*); and a cerebral abscess in a patient with legionellosis (*3*).

*L. feeleii* was first described in 1984 as the causative agent of a Pontiac fever outbreak (*4*). *L. feeleii* was responsible, according to a recent review, for only 10 reported cases of infections, all of which were pneumonia, only 1 complicated by endocarditis (*5*). Unlike lung abscesses, cutaneous lesions caused by *Legionella* spp. are uncommon. Recurrent soft tissue abscesses of the jaw, wrist, and arm caused by *L. cincinnatiensis* were described in a 73-year-old woman with nephrotic syndrome and idiopathic immunoglobulin gammopathy (*6*). *L. micdadei* has been found in a cutaneous abscess of the leg of a 62-year-old immunosuppressed woman, and it was responsible for necrotizing cellulitis that resulted in amputation of the left arm of a recipient of a cadaveric renal transplant (*7*). *L. pneumophila* with mixed flora was identified in a perirectal abscess (*8*) and in skin samples from a patient with lymphoma and cellulitis associated with pneumonia (*9*). The infrequency of reporting *Legionella* spp. cutaneous infections may be explained in part by the fact that *Legionella* spp. agar is not routinely a part of media inoculated for cases of cutaneous abscess. Here we report the identification of *L. feeleii* in a cutaneous infection through the use of a shell vial culture protocol.

In late October 2009, a 66-year-old woman was admitted to Hôpital Nord, Marseille, France, for a papular lesion complicated by cellulitis and an abscess, centered on her right leg (Figure). The patient’s history noted that she had been bitten by an insect or spider (suspected to be a spider) on October 9. The next day, the patient had a fever of 39°C, and a papular lesion appeared around the bite. Four days later, the fever had persisted, and she was given amoxicillin-clavulanate and local wound care. Two days later, the lesion became necrotic, and levofloxacin was added to the medication regimen. At day 10 after the bite, cellulitis with a central abscess appeared on her leg. The patient was transferred to Hôpital Nord. At admission, she had leukocytosis (16.9 × 10^9^ cells/L) with neutropenia (0.51 × 10^9^ cells/L), slight anemia (104 g/dL), and inflammatory syndrome (C-reactive protein 72 mg/L, erythrocyte sedimentation rate 150 mm after 1 h); she was also still febrile (38°C). She had a history of chronic lymphocytic leukemia. A cutaneous biopsy sample showed inflamed and necrotic tissue, which suggested squamous cell evocating carcinoma.

**Figure:**
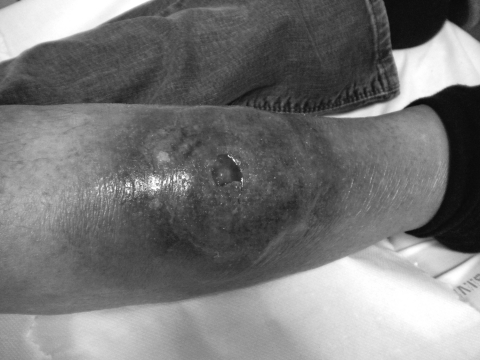
Cellulitis with a central abscess present at time of patient’s admission to hospital, Marseille, France, 2010. A color version of this figure is available online (www.cdc.gov/EID/content/17/1/145-F.htm).

Gram staining of tissue samples did not show any bacteria, and conventional cultures incubated under aerobic and anaerobic conditions did not lead to growth after 3 weeks of incubation. *Je ne sais pas* (or “I don’t know [what I’m growing]” [10]) shell vial culture protocol was done on a skin biopsy sample of the lesion. Culturing was performed by the centrifugation shell vial technique with 3.7 mL human embryonic lung fibroblast cell monolayers (Sterilin, Felthan, UK) inoculated with the skin biopsy sample previously triturated in cell culture medium. Small extra- and intracellular bacilli were observed directly inside the shell vial by using Gimenez and Gram staining. DNA extraction, partial 16S rRNA gene amplification and sequencing, and *mip* and *rpoB* gene amplification and sequencing were done on shell vial supernatant. Partial sequence of 16S rRNA identified a *Legionella* sp. Subsequently, *L. feeleii* was identified with 100% and 96.5% sequence similarity for *mip* and *rpoB* genes, respectively. The biopsy sample was unfrozen and then injected onto *Legionella* spp.*–*buffered charcoal yeast extract agar as the supernatant of the shell vial. No growth was obtained from the biopsy sample, but *L. feelii* was identified similarly from colonies growing on buffered charcoal yeast extract agar plates injected with shell vial supernatant. The patient’s necrotic tissues were surgically excised; a vacuum-assisted closure system was used. Reexamination of the tissue biopsy samples ruled out the diagnosis of carcinoma.

Finally, despite the fact that the shell vial technique requires specialized equipment and trained personnel, this method was performed in a reference center to improve the accuracy of a microbiologic diagnosis and, consequently, the care of the patient in uncommon situations (*10*). This improvement in diagnosis and care was also noted in an unusual *L. pneumophila* infection described by our team (*2*).

In our laboratory, we have been performing the *je ne sais pas* protocol almost routinely since 1996 (*10*). Cell cultures provide supplemental tools to elucidate the cause of microbial diseases when results of PCR and classical agar procedures are negative. Furthermore, this procedure provides a means for the isolation of a wide range of intracellular bacteria, even when little biopsy material is available.
